# Clinical conditions and memory complaints in outpatient elderly

**DOI:** 10.1590/S1980-57642009DN20400015

**Published:** 2008

**Authors:** Regina Miksian Magaldi, Alexandre Leopold Busse, Venceslau Antonio Coelho, Daniel Apolinário, Leonardo da Costa Lopes, Erika Satomi, Juliana Yumi Tison Kasai, Wilson Jacob Filho

**Affiliations:** Serviço de Geriatria do Hospital das Clínicas, Departamento de Clínica Médica, Faculdade de Medicina da Universidade de São Paulo.

**Keywords:** memory, cognition, dementia, elderly

## Abstract

Memory complaints are common in elderly, and may be associated with many clinical problems.

**Objective:**

To identify clinical conditions and possible factors related to memory complaints in elderly outpatients presenting at a tertiary unit.

**Methods:**

Patients with memory complaints and normal cognitive screening tests were submitted to clinical and laboratorial testing. Radiological evaluation was performed as needed for diagnosis.

**Results:**

One hundred and seventy-seven subjects were initially evaluated, 12 were excluded because of poor and inconsistent memory complaints. Of the remainder, seventeen had criteria for dementia diagnosis. Ninety-two (55.4%) had one or more comorbid conditions possibly related to their complaints. Major depression was present in 26.0%. Sixteen patients (9.6%) had vitamin B12 deficiency, 8 were in use of inappropriate medications, and 7 (4.2%) had hypothyroidism. Other conditions diagnosed were: generalized anxiety disorder, obstructive sleep apnea syndrome, hyperparathyroidism, normal pressure hydrocephalus. Three patients had severe hearing loss (in 22 with hearing complaints); one had severe visual impairment (in 22 with visual complaints).

**Conclusions:**

Comprehensive evaluation was able to identify treatable conditions possibly related to memory complaints.

## Abstract (pt)

Queixas de memória são comuns em idosos, podendo estar associadas a diversos problemas clínicos.

**Objetivo:**

Identificar condições clínicas e fatores relacionadas a queixas de memória em pacientes idosos encaminhados a um serviço terciário.

**Métodos:**

Pacientes com queixas de memória e testes de rastreio cognitivo normal foram submetidos a avaliação clínica detalhada e testes laboratoriais, além de avaliação radiológica quando necessário para o diagnóstico.

**Resultados:**

Cento e setenta e sete pacientes foram inicialmente avaliados, sendo 12 excluídos devido a queixas frustras e inconsistentes de memória. Dos restantes, 17 preenchiam critérios clínicos para diagnóstico de demência. Noventa e dois (55,4%) apresentavam uma ou mais condições clínicas possivelmente relacionadas às queixas. Depressão maior estava presente em 26%. Dezesseis pacientes (9,6%) tinham deficiência de vitamina B12, 8 estavam em uso de medicações inapropriadas e 7 (4,2%), hipotireoidismo. Outras condições diagnosticadas foram: transtorno de ansiedade generalizada, síndrome da apnéia obstrutiva do sono, hiperparatireoidismo e hidrocefalia de pressão normal. Três pacientes apresentavam hipoacusia grave (em 22 com queixas auditivas), e 1 apresentava perda visual grave (em 22 com queixas visuais).

**Conclusões:**

Avaliação detalhada pode identificar diversas condições clínicas tratáveis, possivelmente relacionadas às queixas de memória.

Subjective memory complaints (SMC) are common amongst the elderly population. In a meta-analysis of 10 community studies involving a total of 17814 elderly, SMCs were reported by 29.8% of individuals with cognitive deficit, and by 17.4% of healthy controls.^1^ Similar studies that have investigated the prevalence of this type of complaint have found rates ranging from 10% to 88%,^2,3^ where the large difference was largely attributed to the heterogeneity of strategies used in case selection.^4^

The majority of studies select patients through screening, by means of the so-called “stimulated complaint”, whereby the elder is expected to answer the question as whether they have memory problems or not. This approach takes on many forms, ranging from simple questions to standardized questionnaires. This type of screening does not however, reflect the situation found in real clinical practice, and tends to select individuals with little or no risk of current or future cognitive impairment.^5^

Spontaneous SMCs, more specifically those that lead to the patient seeking medical assistance, seem to be more clinically significant. In the PAQUID study, in which a cohort of 1503 elderly subjects were followed for 4 years, SMCs reported spontaneously to the doctor in a primary care setting proved a strong predictor of dementia.^6^ The GuidAge study selected 2854 elderly who were older than 70 years and had spontaneously reported memory problems to their doctors in a primary care setting. Following exclusion of cases of dementia, major depression and generalized anxiety, 55.5% of the elderly sample scored 0.5 on the Clinical Rating Scale (CDR), thus revealing a high prevalence of mild cognitive impairment, and pointing to the need for thorough assessment of patients who spontaneously report SMCs.^7^

A comprehensive evaluation is required in patients seeking assistance for SMCs, in a bid to not only screen for possible cognitive decline, but to also detect potentially reversible factors associated to these impairments. Although the notion of reversible dementia had been increasingly called into question,^8^ the importance of factors which may hamper full cognitive functioning and cause mild impairment should not be overlooked. In a prospective study involving patients referred to a memory clinic, individuals with subjective complaints and those with mild cognitive impairment presented a higher prevalence of reversible causes compared to patients with dementia.^9^

Potentially reversible factors associated to cognitive impairment include: use of sedative drugs or those with anti-cholinergic properties, regular alcohol consumption, depression, cyanocobalamin and/or folic acid deficiency, hypothyroidism, normal pressure hydrocephalus, expansive intracranial lesions, subdural hematomas, obstructive sleep apnea syndrome, and sensory deficits. The majority of these conditions have increased prevalence with aging and often coexist to produce a multi-factorial picture requiring comprehensive and multi-faceted management.

Given these factors are largely easily identifiable by comprehensive examination yet attributed to the most common underlying causes, better characterization of the prevalence of these factors in elderly seeking medical assistance for SMC is paramount for the routine practice of healthcare professionals who attend the geriatric population.

## Objectives

To identify the clinical conditions and potentially reversible factors present in a group of elderly patients with memory complaints and referred to a tertiary health service.

## Casuistic and methods

Patients were referred from the screening program of the Geriatric Service of the Clinicas Hospital, University of São Paulo School of Medicine (HC-FMUSP), from 08/2002 to 08/2007 to the Memory Clinic for the Elderly (AMI). This clinic was set up to assess, diagnose and follow up patients with subjective memory complaints who do not meet criteria for dementia diagnosis. Criteria for referral included age older than 60 years, complaint of memory loss, and normal outcome on cognitive screening tests (Mini Mental State Exam adjusted for schooling).^10^ Exclusion criteria were: diagnosis of dementia, depression or other psychiatric disorders, history of stroke or head trauma within the last year. Patients were assessed according to a protocol which entailed routine clinical anamnesis, demographic data, clinical history, medications in use (particularly medicines with anti-cholinergic, sedative or hypnotic properties), specific questions to characterize the presence and duration of the memory complaint and confirmation or otherwise by a guardian (when available), the existence of compromise in basic and instrumental activities of daily living ^11,12^ in addition to questions pertaining to psychiatric symptoms, sensory impairments, and general physical and neurological examination. All participants were submitted to laboratorial assessment to check for metabolic or hydroelectrolyte disturbances, thyroid dysfunction, vitamin B12 or folic acid deficiency, anemia, renal or hepatic failure, and serology for syphilis. Radiological assessment using computerized tomography scan of the head was carried out whenever necessary, along with other exams based on the diagnosis hypotheses considered for each case. Diagnoses of dementia and psychiatric disorders were based on the criteria from the *Diagnostic and Statistical Manual of Mental Disorders – Fourth Edition* (DSM-IV).^13^ Diagnosis of probable and possible Alzheimer’s disease (AD), as well as probable and possible Vascular Dementia (VD) were reached using the criteria of the *National Institute of Neurological and Communicative Disorders and Stroke – Alzheimer Disease and Related Disorders Association* (NINCDS-ADRDA)^14^ and the *National Institute of Neurological Diseases and Stroke- Association Internationale pour la Recherche et l’Einseignement en Neurosciences (NINDS-AIREN)*,^15^ respectively. All diagnoses and the relevance of laboratory test alterations found were analyzed in consensus by 3 specialists. A descriptive analysis of results was carried out and is shown below.

## Results

One hundred and seventy seven patients were assessed, 124 of whom were women (70.0%). Mean age was 74.7 years (±6.8) and mean schooling 5.7 years (±4.2). Twelve patients who had vague and inconsistent memory complaints were excluded from the analysis. Seventeen patients met the clinical criteria for dementia diagnosis, six of whom were probable Alzheimer’s disease (AD), 10 possible AD and one vascular dementia (VD). The mean number of co-morbidities per patient was 3.1 (±1.5) and for medications in use was 4.1 (±2.5). Complaints were confirmed by a close informant during the consultation in 53.3% of the cases. The mean duration of complaint was 31.8 months (ranging from one to 180 months). Complaint duration was between 12 and 60 months in 104 patients (63.0%). Longer complaint duration (greater than or equal to five years) was observed in 29 patients, five of whom were diagnosed with dementia (one probable AD, three possible AD and one VD), 14 presented with depression, and one suffered from generalized anxiety disorder.

Ninety-two patients (55.4%) presented one or more potentially clinically treatable conditions associated to memory complaints (Table 1).

**Table 1:** Clinical conditions associated to memory complaints in elderly outpatients.

Condition	N (%)
Depression	43 (26.0)
Auditory complaints	22 (13.3)
Severe Hearing impairment	3 (1.8)
Visual complaints	22 (13.3)
Severe visual impairment	1 (0.6)
B12 vitamin deficiency	16 (9.6)
Hypothyroidism	7 (4. 2)
Anxiety	5 (3.0)
Drugs	8 (4. 8)
Anticholinergics	4 (2.3)
Benzodiazepines	3 (1.7)
Sedatives	1 (0.6)
OSAS	2 (1.2)
Hyperparathyroidism	1 (0.6)
Normal pressure hydrocephalus	1 (0.6)
Others*	9 (5.4)

OSAS, obstructive sleep apnea syndrome;

* Other conditions include: Uncontrolled clinical diseases (congestive heart failure, unstable angina, diabetes mellitus and myelodysplasia); lung neoplasia and pancreas metastases (none with lesion to the central nervous system); bipolar mood disorder; amyotrophic lateral sclerosis.

## Discussion

The present casuistic comprised patients whose memory complaint was the main reason for seeking assistance or referral to a tertiary service. As mentioned previously, spontaneous memory complaints appear to have a stronger correlation with actual cognitive impairment, irrespective of the root cause.

In view of increasing calls for early diagnosis of dementia, efforts have been dedicated to proper identification of patients with Mild Cognitive Impairment (MCI), most notably in amnestic forms. Although the definition of the condition remains a source of controversy,^16^ and the increased chances of evolving to dementia is being called into question,^17^ it has been acknowledged that individuals who meet the criteria first defined by Petersen,^18^ and more recently reviewed by international consensus,^19,20^ should be assessed and followed up periodically. There was no consensus in the literature consulted, on the best approach for assessing cognition in these individuals, nor on the best follow up strategy.

The demographic profile found in patients analyzed is consistent with an outpatient casuistic from a Brazilian tertiary hospital, comprised predominantly of women with low level of schooling, a high number of co-morbidities, and in use of several medications.

The diagnosis of 17 cases of dementia was not reached in initial screening. This finding stems from the difficulties in interpreting the MMSE in populations with low level of schooling, and owing to the possibility that the functionality investigation was not carried out systematically during triage assessment, due to the limited time available during the consultation.

Confirmation of the memory complaints with the informant was only possible in 53.3% of the cases. This confirmation is considered important for diagnosing Mild Cognitive Impairment according to current criteria, even more so than the complaint reported by the patient. However, many patients, given their autonomy and functional independence, came unaccompanied to appointments, despite requests for a close family member to be present.

A large variation in duration of memory complaint was found (one to 180 months). Although the concept of Subjective Cognitive Impairment is still upheld, a condition that can exist prior to developing MCI^21^ and which can last up to 15 years, very short or long periods of complaint go against the notion of consistent and progressive memory decline in the last year suggestive of MCI.

Several concomitant clinical conditions which are potentially damaging to cognitive functioning were detected in this casuistic. The definition of what is potentially reversible varies among studies as does its prevalence. Meta-analyses of reversible causes of dementia indicate frequencies of 1% to 30%, with reversibility rates becoming progressively lower.^22-24^ The most commonly reported causes in these reviews include depression, drug intoxication, and metabolic disturbances. A Brazilian study found a prevalence of 8.0% for reversible causes of dementia, where partial remission was achieved in 45% of these cases, and full remission in 9.0%.^25^

Assessments investigating reversible causes of cognitive impairment in patients with mild cognitive impairment or subjective memory complaints have not been carried out in a systematic manner in the literature consulted. A prospective study which assessed 1000 patients referred to a memory clinic, found a 19% rate of reversible primary causes of cognitive symptoms, and 23% rate of potentially reversible concomitant conditions. Whereas only 4% of patients in the subgroup with dementia presented potentially reversible causes, 16% of patients with cognitive impairment without dementia, and 38% of those with subjective memory complaints, had potentially reversible causes. The most frequent significant concomitant conditions found were depression (8%), thyroid disease (4%), B12 deficiency (4%), alcoholism (3%) and epilepsy (2%).^9^ Another study assessed 80 patients referred to a memory clinic, and found laboratorial changes in 32 cases, with thyroid dysfunction being the most frequent finding. However, no correlation was found between the presence of these laboratorial changes and the existence or otherwise of cognitive impairment upon presentation and after one year of follow up.^26^

Depression was the most prevalent condition (26.0%) in this casuistic, the treatment of which has the highest chance of reversing cognitive impairment. The relationship between depression and cognitive impairment has been extensively studied, where the presence of depressive symptoms in cognitively healthy patients has been linked to the highest rate of evolution to mild cognitive impairment and dementia.^27^ There is also evidence that neuropsychiatric symptoms, particularly depression, are frequent in patient with mild cognitive impairment.^28^ The role of depression in these patients however, remains unclear. It is believed that, besides constituting an independent cause of cognitive impairment, depressive symptoms could represent a psychological reaction to cognitive decline or reflect the course of a neurodegenerative process.^29^

Visual and hearing complaints have also been found in a high percentage of cases. Visual problems increase with age, even in patients who wear glasses. It is estimated that in the United States, the prevalence rises from 4% among individuals aged 65 to 74 years, to 16% in those aged 80 to 84 years. Similarly, more than half of individuals older than 60 years are estimated to have impaired hearing.^30^ Although severe dysfunctions have been less frequent, the presence of sensory disturbances has been associated with different clinical outcomes including cognitive decline, which seems to be exacerbated when there is an overlap of impairments. Concerning hearing loss, a recent study conducted in Brazil revealed that the population with Mild Cognitive Impairment presented more hearing complaints and significantly higher hearing thresholds than the population without cognitive complaints, suggesting an association between MCI and hearing loss.^31^

Low levels of vitamin B12 are common among elderly and have been systematically implicated as a cause or factor contributing to cognitive impairment.^32^ Epidemiological studies in populations of industrialized countries have show a deficiency rate of approximately 20% (range 5% to 60%), depending on the definition adopted. The study of Framingham found a 12% prevalence among community elderly.^33^ However, the evidence from the literature consulted is unclear regarding the benefits on cognition of replacement, while meta-analyses in the literature have been unable to demonstrate a significant effect of treatment in patients with or without dementia.^34^ This issue has not yet been properly addressed in individuals with MCI.

Thyroid and parathyroid disorders have been considered rare causes of reversible cognitive decline. The PAQUID was unable to associate hypothyroidism with cognitive impairments in elderly patients from the community, nor demonstrate reversal of dementia pictures in treated patients.^35,36^ However, the efficacy of replacement in patients without dementia has yet to be ascertained. Data on hyperthyroidism is scarce, and despite being cited as a reversible cause of cognitive impairment, calcium metabolism disorders present in this patient group seem more closely related to acute confusional states.

The number of patients found using inappropriate medicines, potentially compromising cognition, was lower than expected in view of the high prevalence of polypharmacy and adverse reactions to drugs commonly found among the elderly. The most common cause of drug-induced cognitive decline is the use of psychoactive medication (such as hypnotics, sedatives, opioids analgesics, antidepressives, anticonvulsants, antiparkinsonians and antipsychotics), all widely used in geriatrics.^37^ A study assessing elderly outpatients reported sleep complaints in 59.3% of patients, and use of medications to sleep in 37.3%.^38^ The possibility of underreporting drug use is acknowledged, in spite of the questions put, bearing in mind the high consumption of unprescribed medication in this age group.^39^ Alcohol abuse was not identified in any of the patients, although this has been previously reported in casuistics with potentially reversible dementia.^9,25^

The increasingly diagnosed and highly reversible Obstructive Sleep Apnea Syndrome (OSAS) was found in 2 patients. A study in non-obese healthy elderly diagnosed sleep apnea in 2.9% of individuals in their 60s, 33.3% in their 70s, and 39.5% in their 80s. Significant oxygen desaturation (less than 85%) was seen in 37% of these “normal” octogenarians.^40^ This condition may lead to the emergence of cognitive symptoms, not only as a result of cardiovascular risk but most likely owing to the chronic hypoxemia condition induced.^41^

Normal pressure hydrocephaly was diagnosed in only one patient, who curiously presented no clinical criteria for dementia diagnosis.

Cognitive assessment in these patients, using neuropsychological tests, was able to differentiate subjects who met criteria for mild cognitive impairment from those presenting only subjective memory complaints. This finding allows comparison among groups for the presence of the conditions outlined. Independently, an active search for these diagnoses seems pertinent.

Although no causal relationship can be established between the factors described in this casuistic and memory complaints, it is possible that these exert some influence on cognitive functioning in the patients. The literature has described ever lower rates of truly reversible causes of dementia, while some authors question the validity of carrying out an in-depth investigation in these patients, given that reversibility albeit partial, is rarely possible in cases already harboring dementia.^8,26^ It is noteworthy that all conditions outlined are easily identifiable through clinical assessment and a few non-invasive exams, without placing the patients at any unnecessary risks or requiring costly tests. Furthermore, many of these easily managed conditions go undertreated in elderly, as is the case for sensory deficits. It is known that in order to achieve optimum management of some of the conditions, a multi-disciplinary approach is required, often only accessible in more complex services.

With a gerontological approach in mind that encompasses comprehensive assessment of problems in the elderly population toward maintaining function and independence, and given the high prevalence of co-morbidities in this age group, clinical investigation of potentially reversible associated conditions is desirable. Notwithstanding the low reversibility of previously established dementia pictures, the correction of additional overlapping factors in patients with mild cognitive impairment or only subjective impairment, can be a determinant of improved long-term evolution, a hypothesis which warrants further investigation in future studies.
